# Pied diabétique: aspects épidémiologique, diagnostique, thérapeutique et évolutif à la Clinique Médico-chirurgicale du CHU Sylvanus Olympio de Lomé

**DOI:** 10.11604/pamj.2018.30.4.14765

**Published:** 2018-05-03

**Authors:** Awalou Mohaman Djibril, Edem Komi Mossi, Agbeko Kodjo Djagadou, Abago Balaka, Toyi Tchamdja, Razak Moukaila

**Affiliations:** 1 Service de Médecine Interne, CHU Sylvanus Olympio de Lomé, Togo; 2 Faculté des Sciences de la Santé, Université de Lomé, Togo; 3 Service de Médecine Interne, CHU Kara, Togo; 4 Faculté des Sciences de la Santé, Université de Kara, Togo

**Keywords:** Pied diabétique, morbidité, Lomé, Diabetic foot, morbidity, Lomé

## Abstract

**Introduction:**

Le pied diabétique est une complication fréquente et grave du diabète. Le but de cette étude est de déterminer le profil épidémiologique, diagnostique, thérapeutique et évolutif du pied diabétique en milieu hospitalier à Lomé (Togo)

**Méthodes:**

Etude rétrospective descriptive réalisée du 1^er^ janvier 2011 au 31 décembre 2015 (5 ans) à la clinique médico-chirurgicale du CHU Sylvanus Olympio de Lomé. Etaient inclus dans l'étude, toute patiente hospitalisée pendant la période d'étude pour pied diabétique.

**Résultats:**

La prévalence du pied diabétique était de 12,90%. L'âge moyen était de 60,74 ans (extrêmes: 39 ans et 86 ans). Le sexe masculin prédominait avec un sexe ratio de 1,38. Le diabète de type 2 était retrouvé chez 88,70% des patients. La durée moyenne d'évolution du diabète était de 11,67 ans (extrême: 1 an et 24 ans). Le point de départ des lésions du pied était un traumatisme générant une plaie surinfectée dans 70,97 % des cas. La gangrène (61,29%) et la nécrose ischémique (12,90%) étaient les principales lésions retrouvées. Le principal facteur étiopathogénique retrouvé était la neuropathie (61,29). La majorité des lésions (61,29%) était classée grade 4 et 5 de Wagner et 51,62% des patients avait bénéficié d'une amputation du membre pelvien.

**Conclusion:**

Les lésions du pied sont fréquentes chez les patients diabétiques à Lomé. La lutte contre ce fléau passe par l'éducation des patients et du personnel soignant et par une prise en charge multidisciplinaire et concertée.

## Abstract (en)

**Background:**

Diabetic foot is a common and severe complication of diabetes. This study aimed to determine the epidemiological, diagnostic, therapeutic and evolutionary features of diabetic foot at a Hospital in Lomé (Togo)

**Methods:**

We conducted a retrospective, descriptive study from 1 January 2011 to 31 December 2015 (5 years) at the Medico-surgical Clinic, University Hospital Sylvanus Olympio in Lomé All patients hospitalized for diabetic foot during the study period were enrolled.

**Results:**

Diabetic foot prevalence was 12.90%. The average age of patients was 60,74 years (ranging from 39 to 86 years). Diabetic foot was predominant in male patients, with a sex ratio of 1.38. Type 2 diabetes was found in 88.70% of patients. The mean duration of diabetes evolution was 11.67 years (ranging from 1 to 24 years). Foot lesions were caused by a trauma resulting in a superinfected sore in 70.97% of cases. Gangrene (61.29%) and ischemic necrosis (12.90%) were the most common lesions. Neuropathy was the main etiopathogenetic factor (61.29). The majority of lesions (61.29%) were classified as Wagner grade 4 and 5 and 51,62% of patients underwent lower limb amputation.

**Conclusion:**

Foot lesions are frequent in diabetic patients living in Lomé. The fight against this scourge depends on education of patients and their caregivers as well on multidisciplinary and concerted patient management.

## Introduction

Le pied diabétique regroupe toute infection, ulcération ou destruction des tissus profonds du pied associées à une neuropathie et/ou une artériopathie périphérique des membres inférieurs chez le diabétique [1]. C'est une complication fréquente et grave du diabète avec un taux d´amputation de membres inférieurs très élevé et des conséquences souvent dramatiques sur le plan socio-économique et psychologique [2]. En Afrique, les lésions du pied chez le diabétique sont malheureusement très courantes. Elles sont à l'origine de 15% à 25% des hospitalisations chez les diabétiques [2,3]. Souvent, la pauvreté, le manque d´hygiène et la marche à pieds nus interagissent pour aggraver l´impact des lésions du pied causées par le diabète [4]. Au Togo, nous disposons de très peu de données sur le pied diabétique [3,5]. L'objectif de cette étude est de décrire le profil épidémiologique, diagnostique, thérapeutique et évolutif du pied diabétique en milieu hospitalier au Togo

## Méthodes

Notre étude s'est déroulée à la Clinique Médico-chirurgicale (CMC) du Centre Hospitalier Universitaire Sylvanus Olympio (CHU-SO) de Lomé qui constitue le centre national de référence. Il s'est agi d'une étude rétrospective descriptive portant sur les dossiers des patients hospitalisés pour pied diabétique du 1er janvier 2011 au 31 décembre 2015, soit une durée de 5 ans. Nous avons désigné par pied diabétique toute infection, ulcération ou destruction des tissus profonds du pied associées à une neuropathie et/ou une artériopathie périphérique des membres inférieurs chez le diabétique. Une fiche d'enquête préétablie comportant les données épidémiologiques (âge, sexe), cliniques (histoire du diabète, facteurs de risque cardiovasculaire, facteur déclenchant les lésions du pied, le type de lésion, la gravité des lésions selon la classification de Wagner et les complications dégénératives), paracliniques, thérapeutiques et évolutives a servi à la collecte des données. Ces données ont été analysées et traitées avec le logiciel statistique «Sphinx 5.3.1.». Les variables quantitatives ont été exprimées en moyenne, et les variables qualitatives en effectif et en pourcentage.

## Résultats

**Aspects épidémiologiques:** L'étude a porté sur 62 patients représentant 12,90% des patients diabétiques hospitalisés à la CMC du CHU-SO pendant notre période d'étude. L'âge moyen était de 60,74 ans avec des extrêmes de 39 ans et 86 ans. Les tranches d'âge les plus représentées étaient celles comprise entre 50 ans et 59 ans (40,30 %) et plus de 70 ans (27,40%). On notait une prédominance masculine (58,10%) avec un sex-ratio de 1,38.

**Figure 1:**
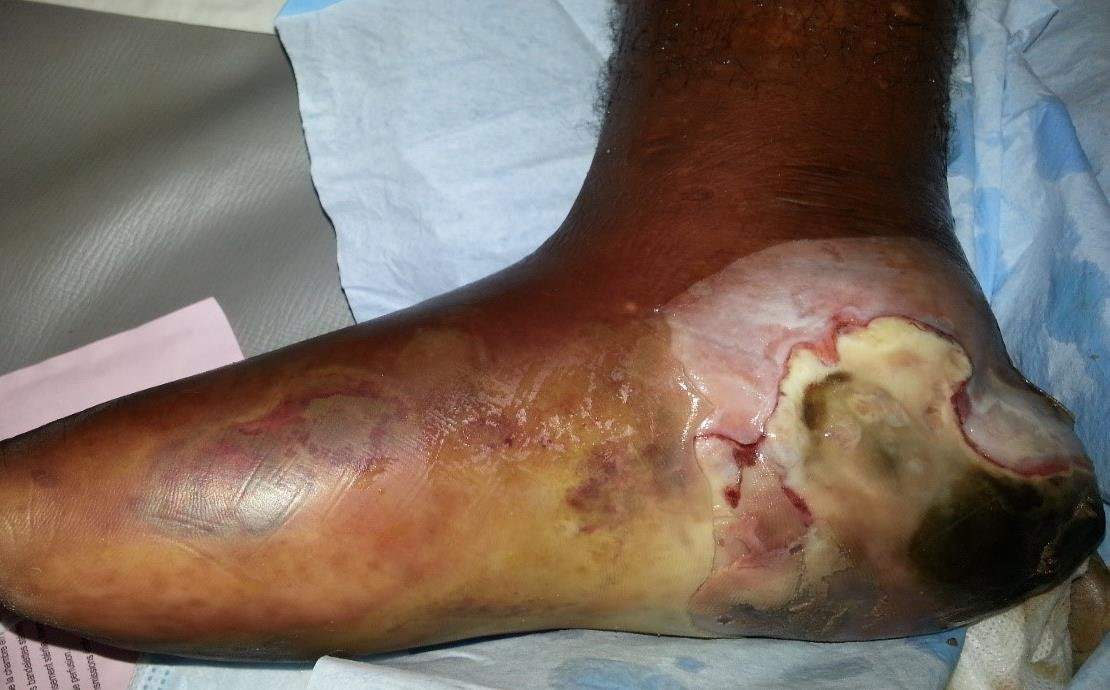
Gangrène infectieuse du pied droit chez un patient diabétique

**Figure 2:**
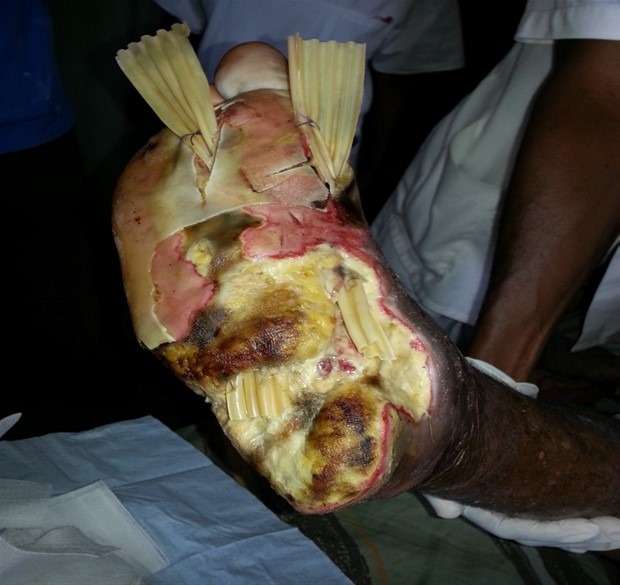
Gangrène infectieuse du pied droit après parage chez un patient diabétique

**Aspects diagnostiques:** La majorité des patients était diabétique de type 2 (88,70%). La durée moyenne d'évolution du diabète était de 11,67 ans. L'hypertension artérielle (HTA) (41,90%), la dyslipidémie (29%) et l'obésité (20,96%) étaient les principaux facteurs de risque cardiovasculaires associés. Presque la totalité des patients (93,50%) présentait au moins une complication dégénérative du diabète dont 77,40% de neuropathie diabétique, 17,7% de rétinopathie diabétique et 3,22% de néphropathie diabétique. Des anomalies électrocardiographiques à type d'hypertrophie ventriculaire et/ou auriculaire, d'ischémie sous-épicardique, de nécrose myocardique, de troubles de rythmes étaient retrouvées chez 61,30%. Le déclenchement des lésions était dû à un traumatisme générant une plaie surinfectée dans 70,97% des cas et à un intertrigo inter orteil (négligé ou mal traité) dans 12,90% des cas. Le délai moyen de consultation des patients était de 33 jours avec des extrêmes de 6 jours et 120 jours. La neuropathie diabétique était retrouvée dans 61,29% des cas, l'artériopathie dans 27,41%, l'association neuropathie-artériopathie dans 35,48%. L'infection quant à elle était au-devant du tableau clinique dans 70,97% des cas. La gangrène infectieuse (61,29%) (Figure 1, Figure 2) et la nécrose ischémique (12,90%) étaient les types de lésion clinique les plus retrouvées (Tableau 1). Les lésions étaient classées grade 4 et 5 de Wagner dans 61,29% (Tableau 2). La glycémie à jeun moyenne à l'admission des patients était de 2,10 g/l avec des extrêmes de 2,10 et 4,11 g/l. L'hémoglobine glyquée moyenne était de 8,65% avec des extrêmes de 7 et 14,2%. Une hyperleucocytose à polynucléaires neutrophiles était notée chez 74,19%. Seul quatorze (14) patients (22,58%) avaient réalisé une radiographie du pied. Et sur ces 14 patients, trois (21,42%) présentaient une ostéite.

**Tableau 1:** Répartition des patients en fonction de l’aspect clinique des lésions

	Effectifs (n)	Pourcentage (%)
Gangrène	38	61,29
Nécrose ischémique	8	12,90
Mal perforant plantaire	7	11,29
Pyodermite	6	9,67
Ulcération	4	6,45
Phlegmon	2	3,23

**Tableau 2:** Répartition des lésions du pied selon la classification de Wagner

	Effectif (n)	Pourcentage (%)
Grade 1	4	6,45
Grade 1 et 2	7	11,29
Grade 3	13	20,97
Grade 4	16	25,81
Grade 5	22	35,48
Total	62	100

**Aspects thérapeutiques et évolutifs:** Le traitement médical a consisté en une antibiothérapie à large spectre chez tous les patients, une insulinothérapie chez 75,81%, la poursuite des antidiabétiques oraux chez 24,19%. L'utilisation des antalgiques et des anticoagulants a été systématique. Les soins locaux étaient réalisés chez tous les patients. Trente-deux patients (19 de sexe masculin et 13 de sexe féminin), soit 51,61% avaient bénéficié d'une amputation du membre pelvien. La gangrène était le principal motif d'amputation (75%). Quatre cas de décès (6,45%) ont été enregistrés. Il s'agissait de deux cas d'embolie pulmonaire post-chirurgie et de deux cas de septicémie. La durée moyenne d'hospitalisation était de 23,11 jours avec des extrêmes de 4 et 92 jours.

## Discussion

La fréquence du pied diabétique à Lomé (12,90%) est comparable à celle observée par Sani au Niger qui a trouvé une fréquence de 13,90% [6]. L'âge moyen dans notre série était de 60,70 ans. Celui rapporté dans la littérature européenne varie entre 67 ans et 73 ans [7]. Les études consacrées aux pieds diabétiques en Afrique ont rapporté une moyenne d'âge inférieure à 60 ans [8,9]. Cette différence d'âge peut s'expliquer par le jeune âge des populations d'Afrique mais surtout par une mauvaise observance thérapeutique par nos patients. Les raisons de cette mauvaise observance sont multiples : la non acceptation du diabète, la tradithérapie, les croyances et surtout la pauvreté [5]. La prédominance masculine parmi la population étudiée est un phénomène que confirment plusieurs auteurs. Sani et al [6] avaient retrouvé un sex-ratio de 2,46; il était de 2,5 pour Amoussou-Guenou [9]. La mauvaise observance thérapeutique généralement reconnue chez les hommes expliquerait cette prédominance masculine [10].

La durée moyenne d'évolution du diabète était de 11,67 ans avec des extrêmes de 1 an et 24 ans. Ceci prouve que les lésions du pied chez le diabétique sont des complications tardives du diabète. Elle était de 13,52 ans à l'Hôpital Militaire d'Instruction Mohammed (HMIM) V de Rabat [11]. Les facteurs de risque cardiovasculaires associés au diabète dans notre série étaient l'HTA suivie de la dyslipidémie, et de l'obésité. Dans l'étude de l'HMIM V de Rabat, seulement 19,20% des patients présentaient une HTA [11]. Une étude cas-témoins réalisée à Tétouan a mis en évidence une association statistiquement significative du pied diabétique avec plusieurs facteurs à savoir : HTA (multiplie le risque de survenue du pied diabétique par 3), la présence d'une complication associée au diabète, le suivi du régime adapté aux patients diabétiques et des facteurs en rapport avec le mode de vie tel que le tabagisme qui multiplierait par trois le risque de survenue du pied diabétique. Tandis que l'association entre la survenue du pied diabétique et la notion de diabète dans la famille, le type de diabète, l'indice de masse corporelle (IMC) et l'alcoolisme n'était pas significative [12]. Le facteur déclenchant des lésions était un traumatisme générant une plaie surinfectée dans 70,97% des cas. Dans la série d'Amoussou-Guenou, les lésions étaient dues aux traumatismes dans 32,86% des cas, aux brûlures dans 2,86% et au port de chaussures inadaptés dans 1,43% des cas [13]. Cliniquement, les lésions du pied étaient dominées par la gangrène (61,29%) et la nécrose ischémique (12,90%). Dans la série de Quassimi, 32,65% des patients présentaient un phlegmon, 28,57% un mal perforant plantaire, 14,28% une nécrose ischémique ; la gangrène était retrouvée dans seulement 8,16% des cas [14]. La sévérité des lésions du pied marquée par une fréquence élevée des lésions de grade 4 et 5 (61,29%) selon la classification de Wagner constitue en soi un risque plus élevé d'amputations majeures. Le mauvais équilibre glycémique de nos patients confirme le fait que l´infection en général, et le pied diabétique en particulier, sont des facteurs de déséquilibre du diabète. La prise en charge du pied diabétique doit être pluridisciplinaire et concertée, impliquant différentes spécialités. Il a été montré que cette approche entraîne une diminution de 49 à 85% du taux d´amputation [1]. La prévalence élevée des amputations dans notre série (51,61%) reflète le degré de gravité des lésions à l'admission. Ce taux est comparable à celui retrouvé au Niger par Sani et al [6]. Le taux de mortalité retrouvé (6,45%) est comparable à celui de Lokrou qui a observé une mortalité de 6,52% [8].

## Conclusion

Les lésions du pied sont fréquentes chez les patients diabétiques à Lomé. Les patients consultent le plus souvent à un stade avancé des lésions compromettant la possibilité de se contenter de gestes de sauvetage du pied. La lutte contre le pied diabétique repose ainsi, d'une part, sur la prévention par l'éducation des patients, du personnel soignant et le dépistage précoce des lésions, et d'autre part, sur une prise en charge multidisciplinaire et concertée

### Etat des connaissances actuelles sur le sujet

- Le pied diabétique est une complication fréquente et grave du diabète;
- Le pied diabétique est la principale cause d'amputation non traumatique chez le diabétique.

### Contribution de notre étude à la connaissance

- Elle apporte des données sur la fréquence du pied diabétique au Togo;
- Les patients consultent à un stade évolué des lésions.

## Conflits d’intérêts

Les auteurs ne déclarent aucun conflit d'intérêts.
